# The DPP-4 inhibitor vildagliptin impacts the gut microbiota and prevents disruption of intestinal homeostasis induced by a Western diet in mice

**DOI:** 10.1007/s00125-018-4647-6

**Published:** 2018-05-25

**Authors:** Marta Olivares, Audrey M. Neyrinck, Sarah A. Pötgens, Martin Beaumont, Nuria Salazar, Patrice D. Cani, Laure B. Bindels, Nathalie M. Delzenne

**Affiliations:** 1 Metabolism and Nutrition Research Group, Louvain Drug Research Institute, Université catholique de Louvain, 73 av. E. Mounier, Box B1.73.11, 1200 Brussels, Belgium; 2 Department of Microbiology and Biochemistry, Instituto de Productos Lácteos de Asturias (IPLA-CSIC), Villaviciosa, Spain; 3 Walloon Excellence in Life sciences and BIOtechnology (WELBIO), Louvain Drug Research Institute, Université catholique de Louvain, Brussels, Belgium

**Keywords:** Antimicrobial peptides, DPP-4 activity, Gut microbiota, Gut–liver axis, Vildagliptin, Western diet

## Abstract

**Aims/hypothesis:**

Dipeptidyl peptidase 4 (DPP-4) inhibitors are agents designed to increase the half-life of incretins. Although they are administered orally, little is known about their effects on the gut microbiota and functions, despite the fact that some bacteria present in the gut microbiota exhibit DPP-4-like activity. Our objective was to study the impact of the DPP-4 inhibitor vildagliptin on gut functions and the intestinal ecosystem in a murine model of obesity induced by a Western diet (WD).

**Methods:**

Twenty seven male C57BL/6J mice were randomised to receive a control diet, a WD (45% kJ from fat and 17% kJ from sucrose) or a WD + vildagliptin (0.6 mg/ml in drinking water) for 8 weeks.

**Results:**

Vildagliptin significantly reduced DPP-4 activity in the caecal content and faeces. Vildagliptin impacted on the composition of the gut microbiota and its metabolic activity. It mainly decreased *Oscillibacter* spp. (a direct effect independent of DPP-4 activity was shown on cultured *O*. *valericigenes*), increased *Lactobacillus* spp. and propionate, and reduced the ligands of Toll-like receptors 2 and 4. Vildagliptin protected against the reductions in crypt depth and ileal expression of antimicrobial peptides induced by the WD. In the liver, the expression of immune cell populations (*Cd3g* and *Cd11c* [also known as *Itgax*]) and cytokines was decreased in the WD + vildagliptin-fed mice compared with the WD-fed group. Ex vivo exposure of precision-cut liver slices to vildagliptin showed that this response was not related to a direct effect of the drug on the liver tissue.

**Conclusions/interpretation:**

Our study is the first to consider the DPP-4-like activity of the gut microbiota as a target of DPP-4 inhibition. We propose that vildagliptin exerts beneficial effects at the intestinal level in association with modulation of gut microbiota, with consequences for hepatic immunity. If relevant in humans, this could open new therapeutic uses of DPP-4 inhibition to tackle gut dysfunctions in different pathophysiological contexts.

**Data availability:**

The sequences used for analysis can be found in the MG-RAST database under the project name MYNEWGUT3.

**Electronic supplementary material:**

The online version of this article (10.1007/s00125-018-4647-6) contains peer-reviewed but unedited supplementary material, which is available to authorised users.

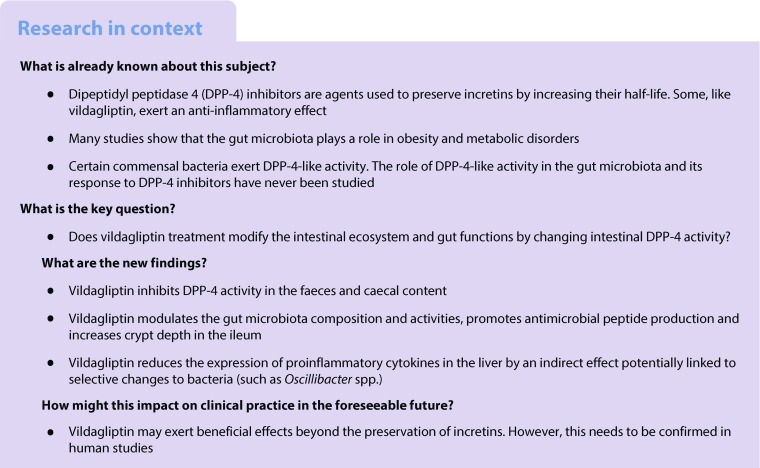



## Introduction

Enteroendocrine cells release incretins in response to nutrient ingestion. The two main incretins—glucagon-like peptide (GLP) 1 and glucose-dependent insulinotropic peptide (GIP)—regulate the postprandial secretion of insulin [1]. One limitation of GLP-1 and GIP activities is their short half-life, because they are rapidly cleaved and inactivated by dipeptidyl peptidase 4 (DPP-4). DPP-4 exists as a membrane-anchored cell surface peptidase and as a soluble form in the circulation [2]. The expression and circulation levels of DPP-4 in people with type 2 diabetes are higher than in those without diabetes [3]. Additionally, DPP-4 is considered an adipokine positively correlated to body weight, adipose tissue inflammation and insulin resistance in obese individuals [4, 5].

Sitagliptin, anagliptin and vildagliptin are a new type of DPP-4 inhibitors which are administered orally and are well tolerated [6]. Although first approved in 2006, their effects on essential intestinal functions remain poorly studied. In 2011, Waget et al described how inhibition of DPP-4 activity in the intestine was involved in regulating glycaemia [7]. Recently, Mulvihill et al demonstrated that glucose tolerance depended on inhibition of DPP-4 activity in haematopoietic and endothelial cells, but there was no involvement of the DPP-4 expressed by enterocytes [8]. DPP-4 activity modulates the functionality of more than 40 potential substrates, including cytokines, chemokines and growth factors, some of which are particularly relevant for gut homeostasis [9]. For example, the preservation of GLP-2 with DPP-4 inhibitors (valine pyrrolidide) has been proposed to stimulate intestinal epithelial growth [10, 11].

Besides cleaving gut peptides, DPP-4 plays a role in the immune response. Soluble DPP-4 increases the expression of Toll-like receptors (TLRs) and activates NFκB signalling, resulting in the secretion of proinflammatory cytokines [12, 13]. These effects can be reduced by DPP-4 inhibitors [14–16]. For instance, in macrophages vildagliptin counteracts the production of cytokines and expression of TLR-2 and TLR-4 induced by soluble DPP-4 and lipopolysaccharide [13]. As TLRs are part of the innate immune response and play a crucial role in the maintenance of gut homeostasis, investigating the effect of vildagliptin in the context of intestinal health may produce particularly relevant results [17].

Vildagliptin is principally absorbed in the small, and to a lesser extent in the large, intestine [18]. During its passage through the intestinal tract, eukaryotic cells as well as the gut microbiota are exposed to vildagliptin. It has been widely described that intestinal microbes respond to environmental factors such as drugs and food, generating protective or detrimental effects [19, 20]. Interestingly, some genera of bacteria in the gut microbiota, such as *Prevotella* and *Lactobacillus*, have been reported to exert DPP-4-like activity [21–23]. Furthermore, some strains of *Bifidobacterium* and *Lactobacillus* can produce DPP-4 inhibitors [24, 25]. To our knowledge, the DPP-4-like activity of the gut microbiota has never been studied. One study (without assessment of DPP-4 activity) reported that vildagliptin caused changes in the gut microbiota and that the changes were more significant in rats fed a high-fat diet than in those fed a control diet [26]. Furthermore, a recent report showed that high-fat, high-sugar diets altered the activation of enteric neurons by GLP-1 regulating insulin secretion, an effect that seemed to be also dependent on the gut microbiota [27]. We therefore assessed the effect of vildagliptin on the gut microbiota and intestinal functions of a murine model of obesity induced by a Western diet (WD), a model that induces gut dysfunction and related hepatic inflammation.

## Methods

### Animals and treatments

Two animal experiments were performed. In experiment 1, 27 male 9-week-old C57BL/6J mice (Janvier Labs, Saint-Berthevin, France) were purchased. Three mice were housed in one individually ventilated cage. In experiment 2, four male 12-week-old C57BL/6J mice (Janvier Labs) were housed in one individually ventilated cage. Mice were kept in a pathogen-free environment with a 12 h daylight cycle and free access to food and water. The acclimatisation period lasted 1 week on a standard diet (AIN-93M; ssniff, Soest, Germany). The experiments were approved by and performed in accordance with the guidelines of the local ethics committee of Université catholique de Louvain. Housing conditions were as specified by the Belgian Law of 29 May 2013 regarding the protection of laboratory animals (Agreement no LA 1230314).

**Fig. 1:**
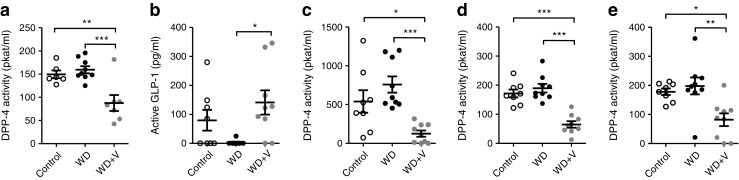
(**a**) DPP-4 activity in the portal vein, (**b**) concentration of active GLP-1 in the portal vein and (**c**) DPP-4 activity in the ileum, (**d**) caecum and (**e**) colon. Mice were fed a control diet, a WD or a WD + vildagliptin (WD+V). Significant differences between conditions are shown as *p < 0.05, **p < 0.01 and ***p < 0.001 according to one-way ANOVA followed by Tukey’s post hoc test

In experiment 1, mice were randomised based on body composition assessed by NMR (LF50 minispec; Bruker, Rheinstetten, Germany) to minimise differences (initial mean body weight ± SEM was 24.46 ± 0.23 g). No blinding procedure was followed. The groups (*n* = 9) were: (1) group-fed a control diet (D12450K; Research Diets, New Brunswick, NJ, USA) containing 10% kJ fat; (2) group-fed a WD (D12451; Research Diets) containing 45% kJ fat and 17% kJ sucrose; and (3) group-fed a WD plus vildagliptin (Cayman, Ann Arbor, MI, USA [supplied by Sanbio, Uden, the Netherlands]) in the drinking water (0.6 mg/ml according to previous studies [13], corresponding to approximately 50 mg kg^−1^ day^−1^). To discriminate the effect of vildagliptin from the effect of the WD on DPP-4 activity, we pretreated with vildagliptin for 2 weeks. In the third week, the WD was introduced. A scheme of the experimental design is shown in electronic supplementary material (ESM) Fig. 1.

After 8 weeks and 6 h of fasting, glycaemia was measured using a glucometer (Roche Diagnostics, Basel, Switzerland) on blood from the tail. Mice were anaesthetised with isoflurane gas (Abbot, Lake Bluff, IL, USA). Portal blood was collected. Mice were necropsied after cervical dislocation. Liver, adipose tissue and caecal content and tissue were weighed. Blood, liver, caecal content and tissue, ileum and colon were collected, frozen in liquid nitrogen and stored at −80°C until analysed.

Details of experiment 2 are given below in the section Precision-cut liver slices.

### Biochemical analysis

Portal active GLP-1 (7-36 amide and 7-37), insulin and total GIP were determined in plasma collected in an EDTA tube with a DPP-4 inhibitor (DDP4-010; Millipore, Darmstadt, Germany) using a multiplex immunoassay kit (Bio-Plex; Bio-Rad, Nazareth, Belgium).

### Analysis of gut microbiota composition

Genomic DNA was extracted from the caecal content using a QIAamp DNA Stool Mini Kit (Qiagen, Hildren, Germany), including a bead-beating step. The composition of the gut microbiota was analysed by Illumina sequencing of the 16S rRNA gene (Illumina, San Diego, CA, USA). Illumina sequencing was performed as previously described [28, 29]. The V5–V6 region of the 16S rRNA gene was amplified by PCR using modified primers. The amplicons were purified, quantified and sequenced using the Illumina MiSeq system to produce 2 × 300 bp sequencing products, at the University of Minnesota Genomics Center. Subsequent bioinformatics and biostatistics analyses were performed as previously described [28]. The full protocol and statistical analyses are described in ESM Methods.

In addition, in the caecal content, some components of the gut microbiota were analysed by quantitative real-time PCR (qPCR) as previously described [28]. Primers are presented in ESM Table 1.

### Gene expression analyses

Total RNA was isolated from tissues and qPCR was performed using the StepOne System (Applied Biosystems, Waltham, MA, USA) to analyse expression of the following genes: *Cd3g*, *Cd11c* (also known as *Itgax*), *Cd68*, *Cd163*, *Claudin2* (also known as *Cldn2*), *DefA*, *F4/80* (also known as *Adgre1*), *Il1b*, *Il6*, *Il10*, *Ki67* (also known as *Mki67*), *Lyz1*, *Mcp1* (also known as *Ccl2*), *Muc2*, *Ocln*, *Pla2g2a*, *Reg3g*, *Tcf4*, *Tnfα* (also known as *Tnf*) and *Zo1* (also known as *Tjp1*). Data were analysed using the 2^-ΔΔCT^ method and expression was normalised to *Rpl19* (see ESM Methods and ESM Table 2 for further details).

### Short-chain fatty acids

Caecal content was diluted 1:6 in LAL reagent water (Lonza, Walkersville, MD, USA) and homogenised with a tissue lyser (Qiagen) for 4 min without beads to avoid bacteria disruption. Samples were centrifuged (8000 *g*, 2 min). Cell-free supernatants were used for the quantification of acetate, butyrate and propionate as previously described [30] and for the analyses of TLR agonists as described below.

### TLR-2 and TLR-4 agonists

TLR-2 and TLR-4 agonists were measured using HEK-Blue reporter cell lines according to the manufacturer’s instructions (InvivoGen, San Diego, CA, USA). Cells were exposed to the supernatants of caecal content and the protocol previously described was followed [31].

### Histological analysis of the intestine

Crypt depth and villus length were measured after H&E staining. Sections were digitised (Leica SCN400; Leica, Wetzlar, Germany) and images were captured using Leica Image Viewer software, version 4.0.4. At least ten measurements per mouse were made.

### Precision-cut liver slices

After 1 week of acclimatisation, mice in experiment 2 were anesthetised with ketamine (100 mg/kg body weight) (Nimatek; Eurovet, Bladel, Netherlands) and xylazine (100 mg/kg body weight) (Rompun; Bayer, Leverkusen, Germany). Precision-cut liver slices (PCLS) were prepared as previously described [32, 33]. PCLS were incubated without (control medium) or with vildagliptin (0.6 mg/ml) in oxygenated and supplemented Waymouth’s medium for 4 h at 37°C under agitation. PCLS were frozen in dry ice until analysed.

### Bacterial growth conditions

Growth curves of *Oscillibacter valericigenes* DSM18026 and *L. reuteri* 100-23 in the presence of vildagliptin (0.6 mg/ml) or absence of vildagliptin (control broth) were determined (ESM Methods).

To measure DPP-4 activity 5 ml of overnight cultures were harvested and processed as described below.

### DPP-4 activity

DPP-4 activity was measured by the cleavage of para-nitroanilide (PNA) from Gly-Pro-PNA (Sigma, St. Louis, MO, USA). Samples (20–50 mg) were suspended in TRIS-base buffer (50 mmol/l, 1% *N*-octylglucoside, pH 8.3), homogenised for 2 min with a tissue lyser and centrifuged (3000 *g*, 20 min). The supernatant (20 μl) was incubated with Gly-Pro-PNA. The enzymatic activity was measured in a kinetic analysis of 30 min at 37°C (380 nm) (SpectraMax M2; Molecular Devices, San Jose, CA, USA) and quantified with a standard curve of free PNA. In tissues and bacterial samples, the values were normalised by the amount of protein (Bradford method).

### Statistical analyses

The number of mice per group was based on previous experiments to measure the primary outcomes (the WD increased the body weight) with the minimum number of animals [34]. Analyses were performed by one-way ANOVA followed by post hoc Tukey’s tests (GraphPad Prism, version 5; GraphPad, La Jolla, CA, USA). For the growth curves, two-way ANOVA followed by Bonferroni’s post hoc test was performed. A *χ*^2^ test was used for categorical data (active GLP-1 and TLR-4 agonist) (Statgraphics Plus, version 5.1; Statgraphics, The Plains, VA, USA). Data of microbiota composition were analysed using Welch’s *t* test and the false discovery rate was applied for *p* value correction (*q* value) according to the Benjamini–Hochberg procedure. Ecological descriptors and data from the PCLS experiment were analysed using Welch’s *t* test. Multiple correlation analyses were performed in R version 3.1 (www.R-project.org), with adjustment of *p* values according to the Benjamini–Hochberg procedure Results were considered statistically significant at *p* < 0.05. Any exclusion decision was supported by Grubbs’ test. Plots were generated using GraphPad showing the mean ± SEM. Each dot represents one biological replicate.

## Results

### Vildagliptin decreased systemic and intestinal DPP-4 activity

Vildagliptin reduced DPP-4 activity in the portal vein and increased the concentration of active GLP-1 (Fig. 1a, b). In addition, vildagliptin significantly reduced DPP-4 activity in the ileum, the caecum and the colon (Fig. 1c–e).

Organ weights and food intake are presented in ESM Table 3. WD-fed mice showed a significant increase in body weight and adiposity. Body weight evolution, glycaemia, insulin and GIP are shown in ESM Fig. 2. Neither WD nor vildagliptin modified the markers related to glucose homeostasis.

### Vildagliptin abolished DPP-4 activity in faeces and caecal content

**Fig. 2:**
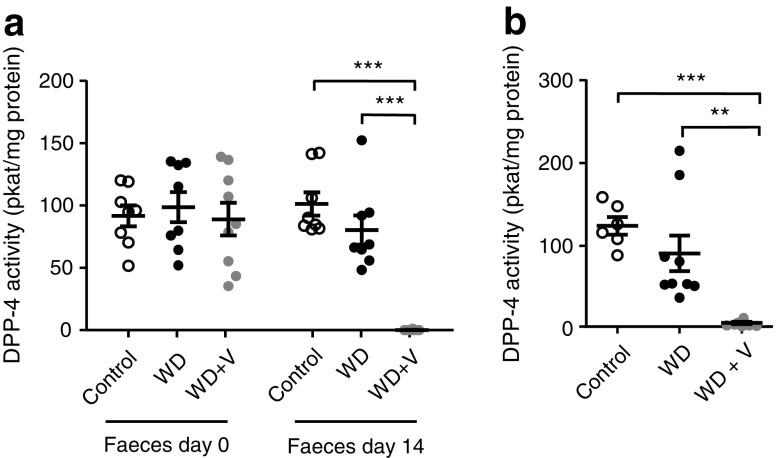
DPP-4 activity (**a**) in the faeces before and after administration of vildagliptin for 2 weeks (14 days) and (**b**) in the caecal content (8 weeks). Mice were fed a control diet, a WD or a WD + vildagliptin (WD+V). Significant differences between conditions are shown as **p < 0.01 and ***p < 0.001 according to one-way ANOVA followed by Tukey’s post hoc test

DPP-4 activity in the faeces did not differ between groups before the introduction of the treatments. The administration of vildagliptin for 2 weeks completely abolished DPP-4 activity in the faeces (Fig. 2a). The effect persisted after the introduction of the WD, as shown by the measurement of DPP-4 activity in the caecal content at the end of the experiment (Fig. 2b).

### Vildagliptin modified gut microbiota composition and activity in vivo and inhibited growth of *O*. *valericigenes* in vitro, independently of DPP-4 activity

**Fig. 3:**
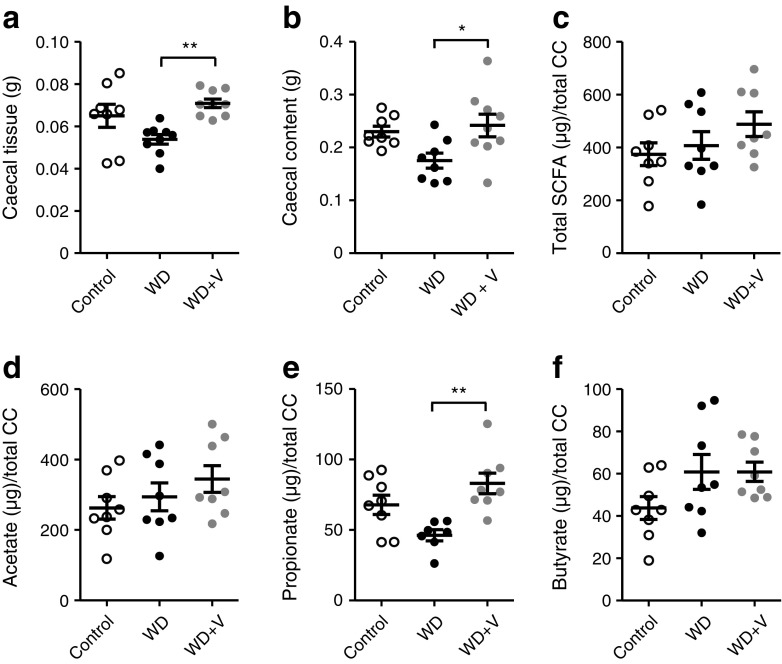
Weight of (**a**) caecal tissue and (**b**) caecal content. (**c–f**) Amount of SCFA in the caecal content (CC): (**c**) total SCFA, (**d**) acetate, (**e**) propionate and (**f**) butyrate. Mice were fed a control diet, a WD or a WD + vildagliptin (WD+V). Significant differences between conditions are shown as *p < 0.05 and **p < 0.01 according to one-way ANOVA followed by Tukey’s post hoc test

Vildagliptin increased the weight of the caecal content and caecal tissue compared with its control group (the WD-fed mice) (Fig. 3a, b). To assess whether these observations were linked to bacterial fermentation, we quantified the production of short-chain fatty acids (SCFA) in the caecal content. Vildagliptin significantly increased the amount of propionate compared with the WD group (Fig. 3e). There were no differences in total amount of SCFA, acetate or butyrate (Fig. 3c, d, f).

**Fig. 4:**
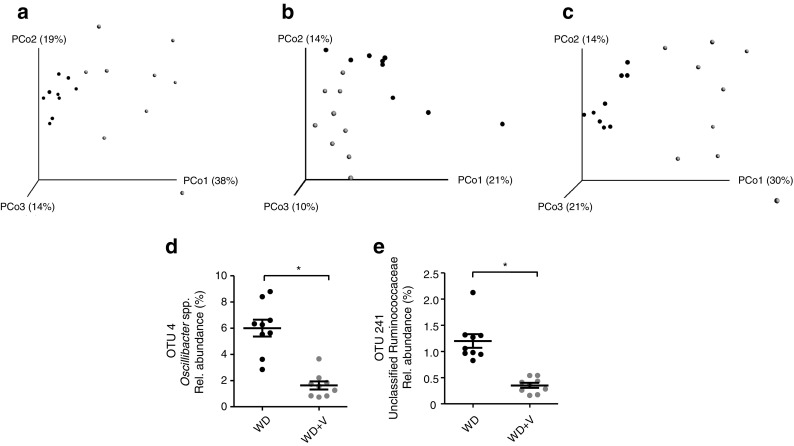
Principal coordinate (PCo) plots of beta diversity based on (**a**) Morisita–Horn, (**b**) binary Jaccard and (**c**) Bray–Curtis indexes. (**d**, **e**) Relative (Rel.) abundance of OTUs modified by vildagliptin assessed by Illumina sequencing in DNA extracted from caecal content. Mice were fed a WD (black symbols) or a WD + vildagliptin (WD+V; grey symbols). Data were analysed using Welch’s *t* test and *p* values were corrected using the Benjamini–Hochberg method. Significant differences are shown as *q < 0.05

We sequenced the 16S rRNA gene of the two WD-fed groups with or without addition of vildagliptin. Principal coordinate analysis (PCoA) of beta diversity based on Morisita–Horn distance showed that vildagliptin accounted for 38% of the variation in the dataset (Adonis method, 1000 permutations; *p* < 0.001). The majority of the variation explained by the treatment was further confirmed by PCoA using binary Jaccard and Bray–Curtis indexes (14% and 26%, respectively; *p* < 0.001) (Fig. 4a–c). No differences were observed in any of the six indexes of alpha diversity analysed (ESM Fig. 3). A total of 293 operational taxonomic units (OTUs) were identified, of which 67 differed significantly between the two groups at the *p* < 0.05 threshold (ESM Table 4). When the false discovery rate was applied for *p* value correction, only two OTUs corresponding to the genus *Oscillibacter* spp. (OTU 4, *p* < 0.001, *q* = 0.016) (Fig. 4d) and unclassified Ruminococcaceae (OTU 241, *p* < 0.001, *q* = 0.016) (Fig. 4e) remained significantly reduced by vildagliptin.

**Fig. 5:**
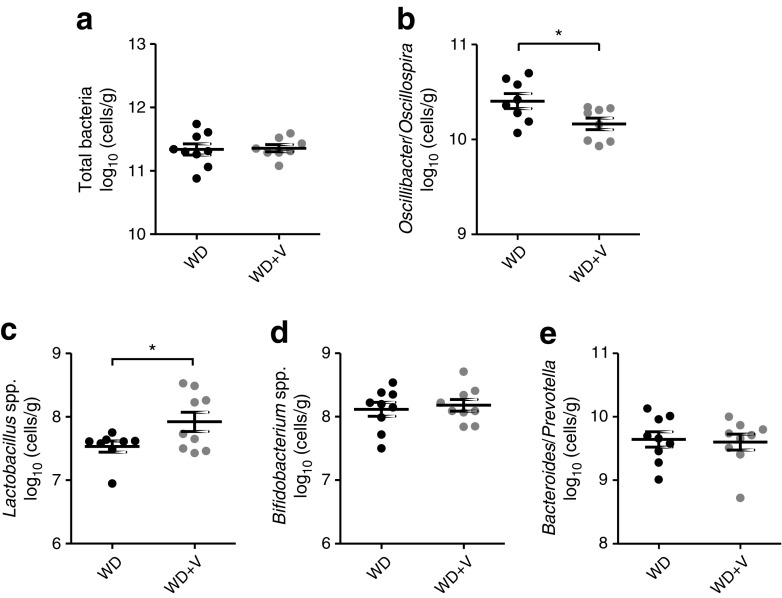
Gut microbiota composition analysed by qPCR in DNA extracted from caecal content. (**a**) Total bacteria, (**b**) *Oscillibacter*/*Oscillospira*, (**c**) *Lactobacillus* spp., (**d**) *Bifidobacterium* spp. and (**e**) *Bacteroides*/*Prevotella*. Mice were fed a WD or a WD + vildagliptin (WD+V). Significant differences are shown as *p < 0.05 according to Welch’s *t* test

Furthermore, some specific bacteria were quantified using qPCR to confirm the reductions in *Oscillibacter* spp. and to quantify the bacteria initially hypothesised to be affected by DPP-4 inhibition. This included bacteria with DPP-4-like activity (*Prevotella* and *Lactobacillus*) [21–23] and those that produce DPP-4 inhibitors (*Bifidobacterium* and *Lactobacillus*) [24, 25]. Vildagliptin caused a significant reduction in *Oscillibacter*/*Oscillospira* and an increase in *Lactobacillus* spp. (Fig. 5). The latter observation is consistent with the sequencing data of *L. johnsonii* that tends to increase with vildagliptin (OTU 177, *p* = 0.016, *q* = 0.155) (ESM Table 4).

**Fig. 6:**
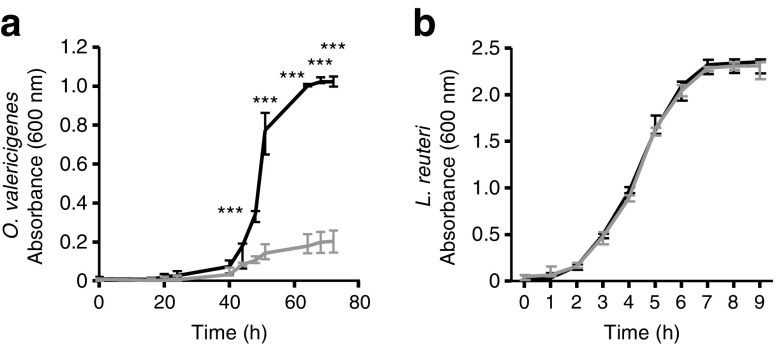
Growth curves of (**a**) *O. valericigenes* and (**b**) *L. reuteri* in control broth (black line) and broth with vildagliptin (grey line). Significant differences between conditions are shown as ***p < 0.001 according to two-way ANOVA followed by Bonferroni’s post hoc test. OD, optical density

To study whether vildagliptin explained these changes, we exposed *O*. *valericigenes* to vildagliptin in vitro and observed that the drug inhibited its growth (Fig. 6a). In parallel, we performed the same test with *L. reuteri*, which is a carrier of DPP-4-like activity in the gut microbiota. *L. reuteri* grew similarly in both conditions (Fig. 6b). No DPP-4 activity was detected in the cell-free extracts of *O*. *valericigenes*. Therefore, the effect of vildagliptin on bacterial growth is not truly dependent on the inhibition of bacterial DPP-4 activity (ESM Fig. 4).

### Vildagliptin reduced TLR ligands in caecal content and restored expression of antimicrobial peptides and crypt depth in ileum

**Fig. 7:**
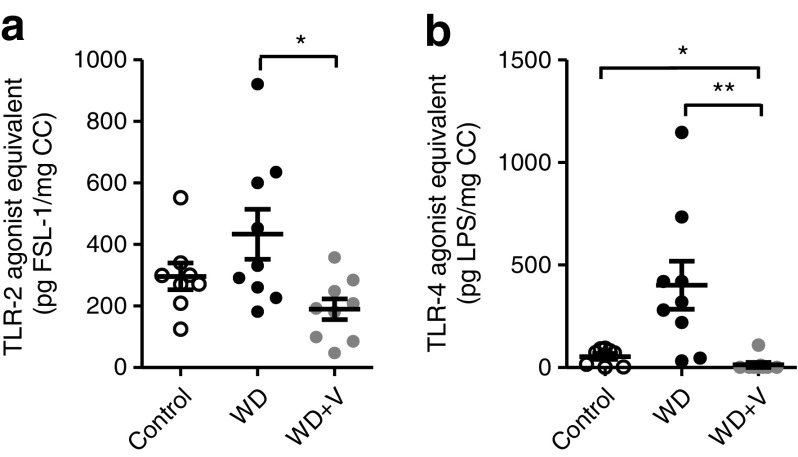
Ligands of (**a**) TLR-2 and (**b**) TLR-4 in caecal content (CC). Mice were fed a control diet, a WD or a WD + vildagliptin (WD+V). Data for TLR-2 were analysed using one-way ANOVA followed by Tukey’s post hoc test. Categorical data (positive/total) for TLR-4 were analysed using the *χ*^2^ test. Significant differences between conditions are shown as *p < 0.05 and **p < 0.01. FSL-1, fibroblast-stimulating lipopeptide; LPS, lipopolysaccharide

The caecal extracts of mice treated with vildagliptin presented lower TLR-2 and TLR-4 ligands compared with those of WD-fed mice (Fig. 7a, b).

**Fig. 8:**
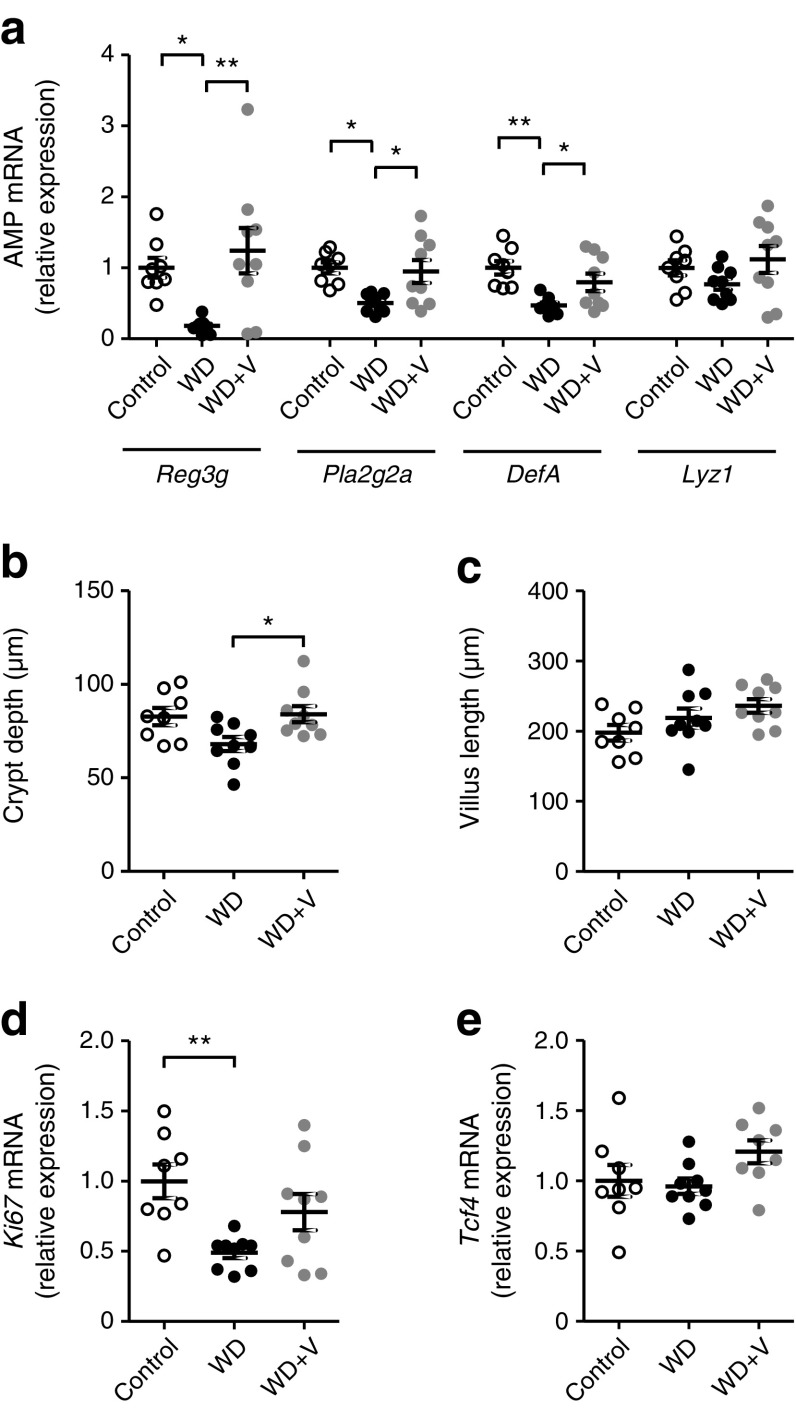
(**a**) Gene expression of AMPs *Reg3g*, *Pla2g2a*, *DefA* and *Lyz1*. (**b**) Crypt depth, (**c**) villus length, (**d**) proliferation marker *Ki67* and (**e**) Paneth cell differentiation marker *Tcf4* in the ileum. Mice were fed a control diet, a WD or a WD + vildagliptin (WD+V). Significant differences between conditions are shown as *p < 0.05 and **p < 0.01 according to one-way ANOVA followed by Tukey’s post hoc test

In the ileum, the expression of antimicrobial peptides (AMPs) such as C-type lectins (*Reg3g*), phospholipases (*Pla2g2a*) and α-defensin (*DefA*) were downregulated in WD-fed mice (Fig. 8a). Vildagliptin counteracted the WD-induced downregulation of *Reg3g*, *Pla2g2a* and *DefA*. We studied whether vildagliptin could also have impacted the morphology and some proliferation markers associated with Paneth cells. Vildagliptin significantly restored crypt depth (Fig. 8b) without changing villus length (Fig. 8c). The histological images are shown in ESM Fig. 5. No differences were observed in *Ki67* or *Tcf4* expression (Fig. 8d, e).

No major changes due to vildagliptin were observed in the expression of markers related to gut barrier function or cytokines; vildagliptin only increased *Claudin2* and *Mcp1* (ESM Fig. 6). In the colon, we analysed some variables previously shown to be affected by vildagliptin in the ileum. Only a significant increase in crypt depth was observed, indicating that the major changes induced by vildagliptin occur in the ileum (ESM Fig. 7).

Finally, we performed a Spearman correlation analysis to evaluate the associations between gut microbes and host variables (ESM Fig. 8). We observed positive correlations between TLR-4 agonists and three OTUs (OTU 4 [*Oscillibacter* spp.], OTU 241 [unclassified Ruminococcaceae] and OTU 40 [*Catabacter* spp.]) and negative correlations between TLR-4 agonists and two OTUs (OTU 117 [unclassified Lachnospiraceae] and OTU 70 [*Parabacteroides goldsteinii*]). Also a positive association was found between *Reg3g* and OTU 16 (unclassified Porphyromonadaceae).

### Vildagliptin indirectly reduced gene expression of proinflammatory cytokines in liver

**Fig. 9:**
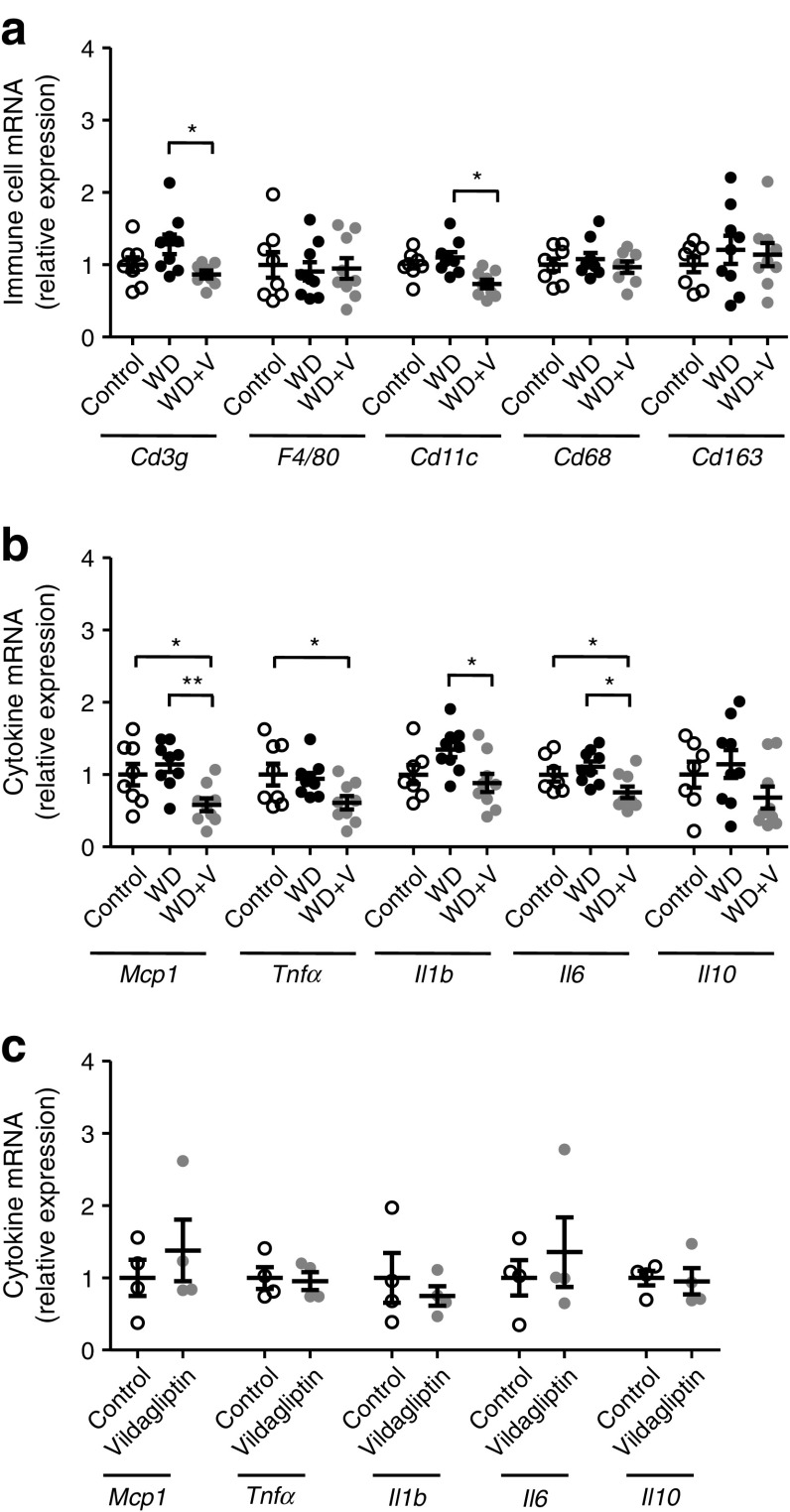
In an in vivo experiment, mice were fed a control diet, a WD or a WD + vildagliptin (WD+V). In an ex vivo experiment, PCLS were exposed to a control medium or medium with vildagliptin. Gene expression of (**a**) biomarkers of immune cell populations, and (**b**) cytokines in the liver in the in vivo experiment and (**c**) cytokines in PCLS in the ex vivo experiment. In (**a**, **b**), significant differences between conditions are shown as *p < 0.05 and **p < 0.01 according to one-way ANOVA followed by Tukey’s post hoc test. In (**c**), data were analysed with Welch’s *t* test

Vildagliptin reduced DPP-4 activity in the portal vein (Fig. 1a), suggesting that the liver is exposed to an efficient dose. Consistent with this, we show that vildagliptin decreased DPP-4 activity in the liver (ESM Fig. 9). Vildagliptin was also associated with a significant reduction in the expression of *Cd3g*, *Cd11c* and the proinflammatory markers *Mcp1*, *Tnfα*, *Il1b* and *Il6* (Fig. 9a, b). To assess whether the changes responded to a direct effect, we performed an ex vivo experiment in which PCLS were exposed to vildagliptin. In contrast to our observations in vivo, no changes in the expression of any cytokines were observed (Fig. 9c).

## Discussion

Inhibition of DPP-4 activity to tackle metabolic disorders through the preservation of incretins has been widely described. However, the additional effects of DPP-4 inhibitors on the intestinal ecosystem remain poorly studied. In the present study, we evaluated whether the DPP-4 inhibitor vildagliptin could impact intestinal homeostasis—including the composition and activity of gut microbiota—using in vivo, ex vivo and in vitro approaches. Our work is the first to consider the DPP-4-like activity of the gut microbiota as a potential target of DPP-4 inhibitors and evaluate, in an animal model of diet-induced obesity, the effect of vildagliptin on gut barrier function, innate immune response and liver. Overall, our results show that vildagliptin improved gastrointestinal function judged by restoration of the expression of AMPs and the depth of the crypts in the ileum. In the liver, vildagliptin was associated with a reduction of cytokine expression. However, the ex vivo experiment with PCLS showed that reductions were not due to a direct effect of the drug, reinforcing the idea that reduced hepatic inflammatory tone might be related to changes at the intestinal level. Furthermore, vildagliptin was associated with changes in beta diversity indexes, reductions in *Oscillibacter* spp. and increases in *Lactobacillus* spp. and caecal propionate levels. Among the 67 OTUs changed by vildagliptin, we can pinpoint some unclassified Lachnospiraceae and Ruminococcaceae, as well as *P. goldsteinii*, as potential contributors to the increase in propionate levels [35], even if no statistically significant correlation backed up this fact. DPP-4 is a multifunctional protein and therefore it is plausible that its inhibition by vildagliptin has induced the broad changes that we observed through several mechanisms. In this regard, we demonstrated three effects of vildagliptin on the intestine, namely: (1) restoration of the active form of gut peptides such as GLP-1; (2) modulation of the innate immune response; and (3) modulation of the gut microbiota. There could be a link between these effects, as illustrated for instance in a study performed in obese prebiotic-fed mice which reported that the gut microbiota influenced the release of substrates of DPP-4 such as GLP-1 and GLP-2 [36]. Furthermore, a high-fat diet can impair enteroendocrine cell function and the release of GLP-1, as confirmed here [37]. We propose that the increase in active GLP-1 in mice treated with vildagliptin can also be partly explained by the increases in propionate in the caecal content [38]. The commutative relationship between microbiota and host is also supported by the fact that AMPs shape the composition of the gut microbiota, and, conversely, the gut microbiota influences host immunity [39].

Since we know that some bacteria exert DPP-4-like activity, one of our outcome measures was to assess whether vildagliptin targets DPP-4 activity of the gut microbiota [21–23]. We have shown for the first time that vildagliptin completely abolished DPP-4 activity in the caecal content and faeces. Analyses of the composition of the gut microbiota showed that *Oscillibacter* spp. was the most striking target of vildagliptin, but its reduction was not explained by DPP-4 inhibition. Only one study has considered the impact of the DPP-4 inhibitors saxagliptin, vildagliptin and sitagliptin on bacterial DPP-4-like activity [40]. Inhibition of the DPP-4-like activity of *Streptococcus mutans* found in the oral microbiota impaired its biofilm formation [40]. None of the DPP-4 inhibitors tested affected the growth of *S*. *mutans* in the concentration range considered (4–2048 μg/ml) [40]. In our study, however, vildagliptin (600 μg/ml) inhibited the growth of *O*. *valericigenes*, even though this bacterium does not exhibit DPP-4 activity. Taken together these results demonstrate the impact of vildagliptin on different members of the microbiota (intestinal and oral) via inhibition of DPP-4-like activity and through a mechanism still unknown. A recent study reported changes in the composition of the gut microbiota by vildagliptin in Sprague–Dawley rat models of diabetes [26]. In agreement with our results, vildagliptin caused a reduction in *Oscillibacter* that was interpreted as a beneficial effect [26]. Furthermore, other animal studies have associated increases in *Oscillibacter* with obese and diabetic phenotypes [41–43], and with increases in intestinal permeability, a condition involved in the development of metabolic disorders [41]. Consequently, vildagliptin induces beneficial changes in the composition of the intestinal microbiota that might become a therapeutic strategy beyond the preservation of incretins.

The gut is populated with multiple types of immune and epithelial cells that work together to maintain tolerance to the gut microbiota and food [17]. The interaction between microbiota and host cells is mediated by TLRs, which are sensors involved in the innate immune response. In addition, a chemical barrier consisting of the secretions of AMPs and other cell products (cytokines and immunoglobulins) is part of the first line of defence [44]. Here, on the one hand, we have shown that vildagliptin reduces levels of TLR-2 and TLR-4 agonists in the caecal content. Previously, it has been reported that vildagliptin suppresses TLR-2 and TLR-4 content in macrophages stimulated with soluble DPP-4 and lipopolysaccharide [13]. This observation is in agreement with the reduction in TLR-4 agonist activity (such as lipopolysaccharide) in the caecal content of mice treated with vildagliptin and demonstrates that vildagliptin impacts the crosstalk between the gut microbiota and the host. On the other hand, vildagliptin also influenced the innate immune response via restoration of AMP expression in the ileum. In agreement with previous studies carried out by our group, a fat-enriched diet caused a reduction in AMPs [34, 45]. Vildagliptin completely counteracted this reduction. We hypothesise that this effect might be linked to GLP-2. As GLP-2 is inactivated by DPP-4, vildagliptin could have preserved its functionality [10, 11]. The role of GLP-2 in inducing antimicrobial products has been shown in GLP-2 receptor knockout mice which had reduced AMPs and impaired mucosal bactericidal activity [46]. Also, the restoration of crypt depth in mice that received vildagliptin could be explained, at least in part, by GLP-2 stimulation of crypt cell proliferation and inhibition of apoptosis [47]. Unfortunately, we could not confirm experimentally this hypothesis due to the lack of commercially available methods to measure active GLP-2.

Finally, we investigated whether the changes observed at the intestinal level could also have impacted the inflammatory tone in the liver. Mice treated with vildagliptin showed a reduction in the expression of lymphocytes (*Cd3g*), macrophage activation (*Cd11c*) and proinflammatory markers. The ex vivo experiment in which PCLS were exposed to vildagliptin showed no changes in any of the inflammatory markers analysed. Even if there is a bias between the in vivo and ex vivo approaches, our observations indicate that the changes induced by vildagliptin at the intestinal level might have also impacted hepatic homeostasis. Specifically, the reinforcement of gut immunity induced by vildagliptin could result in a lower exposure of the liver to microbial stimuli. It would be useful to evaluate how vildagliptin influences gut microbial activity and the gut–liver axis, since microbial metabolites that are prone to reach the liver through the portal vein, such as bile acids (via the farnesoid X receptor) or SCFA (like butyrate), modulate hepatic inflammation and thereby the occurrence of non-alcoholic fatty liver disease [48, 49].

For our study, we selected vildagliptin for its potential effect on inflammation (reduction of TLR expression) [14]. The pharmacokinetics of vildagliptin is different from that of the other DPP-4 inhibitors (being more lipophilic). For instance, the percentage of vildagliptin excreted in the faeces is 4.5%, whereas for sitagliptin and saxagliptin it is reported to be 13% and 22%, respectively [50]. Therefore, the effects of other gliptins on the microbiota—and related metabolic effects—could be relevant, since they are found in higher quantities in the lower intestine. Human data may confirm this hypothesis.

In conclusion, we show that in WD-fed mice presenting gut disorders, vildagliptin affected the composition of the gut microbiota and improved intestinal homeostasis. Due to the multifunctional roles of DPP-4, further studies are needed to decipher the precise molecular mechanisms. If the beneficial effect of vildagliptin on the intestinal tract is confirmed in human studies, it might represent a novel mechanism of vildagliptin to improve human health beyond its standard use.

## Electronic supplementary material

ESM (PDF 775 kb)

## Data Availability

The data that support the findings of this study are available from the corresponding author upon reasonable request. The sequences used for analysis can be found in the MG-RAST database under the project name MYNEWGUT3.

## Abbreviations

AMP: Antimicrobial peptide
DPP-4: Dipeptidyl peptidase 4
GIP: Glucose-dependent insulinotropic peptide
GLP: Glucagon-like peptide
OTU: Operational taxonomic unit
PCLS: Precision-cut liver slices
PCoA: Principal coordinate analysis
PNA: Para-nitroanilide
qPCR: Quantitative real-time PCR
SCFA: Short-chain fatty acids
TLR: Toll-like receptor
WD: Western diet
