# Supplementary material for: The DPP-4 inhibitor vildagliptin impacts the gut microbiota and prevents disruption of intestinal homeostasis induced by a Western diet in mice
Source: Diabetologia. 2018 May 25;61(8):1838–48. doi: 10.1007/s00125-018-4647-6 (PMC6061172; doi:10.1007/s00125-018-4647-6)
Supplement: Supplementary file 1 — (PDF 775 kb) [file 125_2018_4647_MOESM1_ESM.pdf]

Electronic supplementary material (ESM) Methods

Analysis of gut microbiota composition

Amplicon sequencing of the caecal microbiome was done at the University of Minnesota Genomics Center, as previously described by (Gohl *et al*., 2016). The V5-V6 region of the 16S rRNA gene was PCR-enriched using the primer pair V5F_Nextera (TCGTCGGCAGCGTCAGATGTGTATAAGAGACAG**RGGATTAGATACCC**) and V6R_Nextera (GTCTCGTGGGCTCGGAGATGTGTATAAGAGACAG**CGACRRCCATGCANCACCT**) in a 25 µl PCR reaction containing 5 µl of template DNA, 5 µl of 2X HotStar PCR master mix, 500 nM of final concentration of primers and 0.025 U/µl of HostStar Taq+ polymerase (QIAGEN). PCR-enrichment reactions were conducted as follows, an initial denaturation step at 95°C for 5 min followed by 25 cycles of denaturation (20 s at 98°C), annealing (15 s at 55°C), and elongation (1 min at 72°C), and a final elongation step (5 min at 72°C).

Next, the PCR-enriched samples were diluted 1:100 in water for input into library tailing PCR. The PCR reaction was analogous to the one conducted for enrichment except with a KAPA HiFi Hot Start Polymerase concentration of 0.25 U/µl, while the cycling conditions used were as follows, initial denaturation at 95°C for 5 min followed by 10 cycles of denaturation (20 s at 98°C), annealing (15 s at 55°C), and elongation (1 min at 72°C), and a final elongation step (5 min at 72°C). The primers used for tailing are the following: F-indexing primer AATGATACGGCGACCACCGAGATCTACAC[i5]TCGTCGGCAGCGTC and R-indexing primer CAAGCAGAAGACGGCATACGAGAT[i7]GTCTCGTGGGCTCGG, where [i5] and [i7] refer to the index sequence codes used by Illumina.

The resulting 10 µl indexing PCR reactions were normalized using a SequalPrep normalization plate according to the manufacturer's instructions (Life Technologies). 20 µl of each normalized sample was pooled into a trough, and a SpeedVac was used to concentrate the sample pool down to 100 µl. The pool was then cleaned using 1X AMPureXP beads and eluted in 25 µl of nuclease-free water. The final pool was quantitated by QUBIT (Life Technologies) and checked

on a Bioanalyzer High-Sensitivity DNA Chip (Agilent Technologies) to ensure correct amplicon size. The final pool was then normalized to 2 nM, denatured with NaOH, diluted to 8 pM in Illumina's HT1 buffer, spiked with 20% PhiX, and heat denatured at 96C for 2 minutes immediately prior to loading. A MiSeq 600 cycle v3 kit was used to sequence the pool.

Subsequent bioinformatics and biostatistics analyses were performed as previously described Bindels et al, 2016. Initial quality filtering of the reads was performed with the Illumina Software, yielding an average of 106700 pass-filter reads per sample. Quality scores were visualized with the FastQC software (http://www.bioinformatics.babraham.ac.uk/publications.html), and reads were trimmed to 220 bp (R1) and 200 bp (R2) with the FASTX-Toolkit (http://hannonlab.cshl.edu/fastx_toolkit/). Next, reads were merged with the merge-illumina-pairs application v1.4.2 (with P = 0.03, enforced Q30 check, perfect matching to primers which are removed by the software, and otherwise default settings including no ambiguous nucleotides allowed) (Eren *et al*., 2013). For all samples, a subset of 27000 reads was randomly selected using Mothur v1.25.0 (Schloss *et al*., 2009) to avoid large disparities in the number of sequences.

Subsequently, the UPARSE pipeline implemented in USEARCH v7.0.1001 (Edgar, 2013) was used to further process the sequences. Putative chimeras were identified against the Gold reference database and removed. Clustering was performed with 97% similarity cutoff to designate Operational Taxonomic Units (OTUs). A representative sequence of each OTU was used for taxonomy assignment with the RDP seqmatch tool (Cole *et al*., 2014).

The phylotypes were computed as percent proportions based on the total number of sequences in each sample. Alpha diversity indexes and beta diversity indexes were calculated using QIIME (Caporaso *et al*., 2010). PCoA plot of the beta-diversity indexes were visualized using EMPeror (Vazquez-Baeza *et al*., 2013).

Gene expression analyses

Total RNA was isolated from tissues using the TriPure isolation reagent kit (Roche Diagnostics, Germany). Complementary DNA was prepared by reverse transcription of 1 µg of total RNA using the kit Reverse Transcription System (Promega, Madison, WI, USA). Real-time PCR was performed with the StepOne System (Applied Biosystems, The Netherlands). Samples were run in duplicate and the data were analyzed using the $2^{-\Delta\Delta CT}$ method. The expression of the targeted gene was normalized with the expression of the ribosomal protein L19 (*Rpl19*).

Bacterial growth conditions

Oscillibacter valericigenes DSM18026 and *Lactobacillus reuteri* 100-23 were grown in anaerobic conditions in Brain Heart infusion (BHI) (Laborimpex, Brussels, Belgium) and De Man Rogosa and Sharpe (MRS) (Sigma, St Louis, Mo, USA) broths, respectively. To build the curve of growth, fresh media with vildagliptin (0.6 mg/ml) or not (control broth) were inoculated with an overnight cell culture. Periodical values (absorbance at 600 nm) until reaching the stationary phase were registered and plotted *versus* time. Each condition was tested in four independent replicates.

References

Gohl, D.M., *et al.* Systematic improvement of amplicon marker gene methods for increased accuracy in microbiome studies. Nat Biotechnol, 2016;34:942-9.

Bindels, L.B., *et al*. Synbiotic approach restores intestinal homeostasis and prolongs survival in leukaemic mice with cachexia. ISME J, 2016;10:1456-70.

Eren, A.M., *et al*. A filtering method to generate high quality short reads using illumina paired-end technology. PLoS One, 2013;8:e66643.

Schloss, P.D., *et al.* A high-throughput DNA sequence aligner for microbial ecology studies. PLoS One, 2009;4:e8230.

Edgar, R.C. UPARSE: highly accurate OTU sequences from microbial amplicon reads. Nat Methods, 2013;10(10):996-8.

Cole, J.R., *et al*. The Ribosomal Database Project (RDP-II): sequences and tools for high-throughput rRNA analysis. Nucleic Acids Res, 2005;33(Database issue):D294-6.

Caporaso, J.G., *et al*. QIIME allows analysis of high-throughput community sequencing data. Nat Methods, 2010;7:335-6.

Vázquez-Baeza, Y., *et al.* EMPeror: a tool for visualizing high-throughput microbial community data. Gigascience, 2013;2:16.

Benjamini, Y., *et al*. Controlling the False Discovery Rate: a Practical and Powerful Approach to Multiple Testing. J.R. Statist.Soc.B, 1995;57:289-300.

ESM Table 1

Group- or genus-specific 16S-targeted primers for qPCR.

	Forward primer	Reverse primer	Reference
**Total bacteria**	ACTCCTACGGGAGGCAGCAG	ATTACCGCGGCTGCTGG	Ovreas *et al*., 1997
Oscillibacter/Oscillospira	GGCAATGGGCGCAAGCCTGACC	ATTCGTCAGGTACCGTCTTCTRCTC	Walker *et al*., 2011
***Lactobacillus* spp.**	AGCAGTAGGGAATCTTCCA	CACCGCTACACATGGAG	Rinttila *et al*., 2004
***Bifidobacterium* spp.**	GATTCTGGCTCAGGATGAACGC	CTGATAGGACGCGACCCCAT	Gueimonde *et al.,* 2004
Bacteroides/Prevotella	GGTGTCGGCTTAAGTGCCAT	CGGA(C/T)GTAAGGGCCGTGC	Rinttila *et al.,* 2004

References

Ovreas, L., *et al*. Distribution of bacterioplankton in meromictic Lake Saelenvannet, as determined by denaturing gradient gel electrophoresis of PCR-amplified gene fragments coding for 16S rRNA. Appl.Environ.Microbiol, 1997;63:3367-73.

Walker, A.W., *et al*. Dominant and diet-responsive groups of bacteria within the human colonic microbiota. ISME, 2011;5:220-30.

Rinttila, T., *et al*. Development of an extensive set of 16S rDNA-targeted primers for quantification of pathogenic and indigenous bacteria in faecal samples by real-time PCR. J Appl Microbiol, 2004;97:1166-77.

Gueimonde, M., *et al*. New real-time quantitative PCR procedure for quantification of bifidobacteria in human fecal samples. Appl Environ Microbiol, 2004;70:4165-9.

ESM Table 2

Primer sequences for gene expression analyses by RT-qPCR

	Forward primer	Reverse primer
Cd3g	TCTCTACTGGGCTCTCTCCAA	CCATCTCCAAGGAAACCAAC
Cd11c	ACGTCAGTACAAGGAGATGTTGGA	ATCCTATTGCAGAATGCTTCTTTACC
Cd68	CTTCCCACAGGCAGCACAG	AATGATGAGAGGCAGCAAGAGG
Cd163	GGCAACAAATACGTGGCTCT	ATGGGATTTCTCCTCCAACC
Claudin2	AAGGTGCTGCTGAGGGTAGA	AGTGGCAGAGATGGGATTTG
DefA	GGTGATCATCAGACCCCAGCATCAGT	AAGAGACTAAAACTGAGGAGCAGC
F4/80	TGACAACCAGACGGCTTGTG	CAGGCGAGGAAAAGATAGTGT
Il1b	TCGCTCAGGGTCACAAGAAA	CATCAGAGGCAAGGAGGAAAAC
Il6	ACAAGTCGGAGGCTTAATTACACAT	TTGCCATTGCACAACTCTTTTC
Il10	GCTCTTACTGACTGGCATGAG	CGCAGCTCTAGGAGCATGTG
Ki67	CAGACTTGCTCTGGCCTACC	GGTTGGCGTTTCTCCTCTTT
Lyz1	GCCAAGGTCTACAATCGTTGTGAGTTG	CAGTCAGCCAGCTTGACACCACG
Mcp1	GCAGTTAACGCCCCACTCA	CCCAGCCTACTCATTGGGATCA
Muc2	ATGCCCACCTCCTCAAAGAC	GTAGTTTCCGTTGGAACAGTCAA
Oclun	ATGTCCGGCCGATGCTCTC	TTTGGCTGCTCTTGGGTCTGTAT
Pla2g2a	AAGGATCCCCCAAGGATGCCAC	CAGCCGTTTCTGACAGTTCTGG
Reg3g	TTCCTGTCCTCCATGATCAAA	CATCCACCTCTGTTGGGTTC
Rpl19	GAAGGTCAAAGGGAATGTGTTCA	CCTTGTCTGCCTTCAGCTTGT
Tcf4	ATGGCAAACAGAGGAACTGG	GCCTGCTGAGAGTGAAGGAG
Tnfα	AGCCCCCAGTCTGTATCCTT	GGTCACTGTCCCAGCATCTT
Zo1	TTTTTGACAGGGGGAGTGG	TGCTGCAGAGGTCAAAGTTCAAG

ESM Table 3

Body weight gain, total food intake and organ weight.

	Control	WD	WD + vildagliptin
Body weight (BW) gain	3.98 ± 0.31[a]	8.02 ± 0.62[b]	7.47 ± 0.75[b]
Total food intake (g)	176.48 ± 4.06	168.79 ± 1.94	175.84 ± 9.00
Liver (g/100 g BW)	3.72 ± 0.10	3.52 ± 0.03	3.67 ± 0.17
Adipose tissues (g)			
-visceral	0.20 ± 0.02[a]	0.38 ± 0.06[b]	0.32 ± 0.04[ab]
-epididymal	0.52 ± 0.06[a]	1.00 ± 0.15[b]	0.81 ± 0.12[ab]
-subcutaneous	0.40 ± 0.03[a]	0.84 ± 0.10[b]	0.56 ± 0.10[ab]

Mice were fed a control diet, a WD or WD + vildagliptin. Data are expressed as the mean ± SEM. Data with different superscript letters are significantly different at $p<0.05$ according to the one-way ANOVA followed by Tukey's post hoc test.

ESM Table 4. Relative abundance of the OTUs whose levels were affected by vildagliptin (in % of total sequence number).

OTUs	Identification	Identity Score	MEAN		SEM		Welch's *t*-test	
			WD	WD+V	WD	WD+V	*p* value	q value
**OTU_4**	*Oscillibacter* spp.	1.000	6.00514	1.63058	0.64618	0.30584	0.00006	**0.01569**
**OTU_241**	Unclassified Ruminococcaceae	0.941	1.19986	0.35291	0.12987	0.04756	0.00011	**0.01569**
**OTU_117**	Unclassified Lachnospiraceae	1.000	0.01167	0.14978	0.00272	0.02645	0.00077	0.06097
**OTU_76**	Unclassified Ruminococcaceae	1.000	0.12404	0.02994	0.01880	0.00644	0.00083	0.06097
**OTU_25**	*Oscillibacter* spp.	1.000	4.40704	1.94689	0.53590	0.20568	0.00149	0.06319
**OTU_269**	Unclassified Ruminococcaceae	0.962	0.38469	0.15652	0.04925	0.02803	0.00151	0.06319
**OTU_70**	*Parabacteroides goldsteinii*	1.000	0.02427	0.15035	0.00427	0.02697	0.00151	0.06319
**OTU_75**	*Clostridium* spp.	1.000	0.12460	0.03031	0.02163	0.00709	0.00214	0.07846
**OTU_105**	Unclassified Porphyromonadaceae	1.000	0.06168	0.01802	0.00936	0.00817	0.00295	0.07881
**OTU_26**	*Bacteroides goldsteinii*	1.000	0.15239	1.01933	0.03965	0.21171	0.00332	0.07881
**OTU_55**	*Alistipes* spp.	1.000	0.11116	0.26224	0.00957	0.03706	0.00332	0.07881
**OTU_2**	*Clostridium* spp.	0.922	8.71471	3.14125	1.13832	1.15782	0.00342	0.07881
**OTU_18**	*Barnesiella* spp.	0.988	0.47794	2.27240	0.10715	0.44354	0.00350	0.07881
**OTU_65**	Unclassified Lachnospiraceae	1.000	0.25496	0.06846	0.04490	0.03661	0.00559	0.11635
**OTU_232**	Unclassified Lachnospiraceae	0.971	0.00328	0.01879	0.00116	0.00421	0.00596	0.11635
**OTU_49**	Unclassified Porphyromonadaceae	1.000	0.38650	0.15139	0.05591	0.05288	0.00758	0.13258
**OTU_109**	*Clostridium* IV spp.	1.000	0.10657	0.06341	0.01227	0.00602	0.00852	0.13258
**OTU_119**	Unclassified Lachnospiraceae	1.000	0.06546	0.00340	0.01796	0.00157	0.00859	0.13258
**OTU_102**	Unclassified Ruminococcaceae	1.000	0.00651	0.18611	0.00222	0.05195	0.00860	0.13258
**OTU_167**	Unclassified Firmicutes	1.000	0.00429	0.01701	0.00164	0.00376	0.01013	0.14592
**OTU_27**	*Anaerotruncus colihominis*	0.909	0.91565	0.40348	0.12280	0.12834	0.01082	0.14592
**OTU_163**	Unclassified Lachnospiraceae	1.000	0.02174	0.00426	0.00525	0.00219	0.01096	0.14592
**OTU_134**	Unclassified Lachnospiraceae	0.858	0.00692	0.04852	0.00491	0.01266	0.01152	0.14674

ESM Table 4. Continued

OTUs	Identification	Identity Score	MEAN		SEM		Welch's *t*-test	
			WD	WD+V	WD	WD+V	*p* value	q value
**OTU_237**	Unclassified Ruminococcaceae	1.000	0.00097	0.00836	0.00064	0.00229	0.01220	0.14896
**OTU_164**	*Clostridium* spp.	0.975	0.01612	0.03886	0.00449	0.00678	0.01435	0.15502
**OTU_156**	*Lachnospiracea incertae sedis* spp.	1.000	0.02251	0.00258	0.00641	0.00135	0.01446	0.15502
**OTU_100**	Unclassified Lachnospiraceae	1.000	0.12711	0.04475	0.02558	0.01405	0.01495	0.15502
**OTU_177**	*Lactobacillus johnsonii*	0.971	0.00050	0.02629	0.00050	0.00842	0.01550	0.15502
**OTU_114**	*Acetatifactor* spp.	1.000	0.15891	0.01452	0.04721	0.00356	0.01562	0.15502
**OTU_11**	Unclassified Ruminococcaceae	0.905	1.70876	8.07667	0.28270	2.08912	0.01587	0.15502
**OTU_193**	Unclassified Lachnospiraceae	0.971	0.00046	0.00921	0.00046	0.00292	0.01709	0.15720
**OTU_63**	Unclassified Lachnospiraceae	0.848	0.16183	0.06850	0.01895	0.02886	0.01731	0.15720
**OTU_273**	*Barnesiella* spp.	0.941	0.27681	0.96727	0.07647	0.23095	0.01806	0.15720
**OTU_39**	Unclassified Lachnospiraceae	0.920	0.07729	0.54868	0.02140	0.16046	0.01882	0.15720
**OTU_124**	*Clostridium* spp.	0.942	0.08212	0.03193	0.01441	0.01267	0.01894	0.15720
**OTU_62**	Unclassified Lachnospiraceae	1.000	0.49357	0.18885	0.10262	0.04780	0.02051	0.15720
**OTU_104**	Unclassified Lachnospiraceae	1.000	0.13456	0.02593	0.03754	0.01054	0.02065	0.15720
**OTU_165**	Unclassified Porphyromonadaceae	0.913	0.00332	0.02870	0.00139	0.00882	0.02073	0.15720
**OTU_199**	*Enterorhabdus mucosicola*	0.962	0.01086	0.00251	0.00286	0.00114	0.02092	0.15720
**OTU_179**	Unclassified Lachnospiraceae	0.950	0.01640	0.00190	0.00506	0.00144	0.02165	0.15858
**OTU_133**	*Clostridium* XlVa spp.	0.971	0.01265	0.05988	0.00328	0.01678	0.02296	0.16141
**OTU_82**	*Christensenella* spp.	0.743	0.02754	0.12112	0.01100	0.03300	0.02314	0.16141
**OTU_268**	Unclassified Ruminococcaceae	0.913	0.25997	0.09713	0.05871	0.01667	0.02505	0.17070
**OTU_40**	*Catabacter* spp.	0.971	0.75378	0.06639	0.25142	0.01101	0.02570	0.17112
**OTU_73**	*Clostridium* spp.	0.917	0.03021	0.14659	0.01088	0.04314	0.02797	0.17514
**OTU_190**	Unclassified Porphyromonadaceae	1.000	0.02039	0.00423	0.00590	0.00243	0.02850	0.17514
**OTU_89**	*Anaerovorax* spp.	1.000	0.04654	0.08240	0.00601	0.01298	0.02868	0.17514
**OTU_41**	*Bilophila* spp.	1.000	0.34475	0.18569	0.05919	0.01653	0.02869	0.17514

ESM Table 4. Continued

OTUs	Identification	Identity Score	MEAN		SEM		Welch's *t*-test	
			WD	WD+V	WD	WD+V	*p* value	q value
**OTU_33**	Unclassified Porphyromonadaceae	1.000	0.60874	0.28571	0.10998	0.07633	0.02983	0.17754
**OTU_129**	*Butyricicoccus* spp.	1.000	0.10123	0.05905	0.01374	0.01112	0.03030	0.17754
**OTU_43**	*Clostridium* spp.	0.902	0.02109	0.50958	0.00871	0.18872	0.03222	0.18232
**OTU_79**	Unclassified Lachnospiraceae	1.000	0.12667	0.04202	0.03249	0.00805	0.03236	0.18232
**OTU_120**	*Peptococcus* spp.	1.000	0.04859	0.01520	0.01214	0.00704	0.03360	0.18275
**OTU_87**	Unclassified Ruminococcaceae	1.000	0.00425	0.11964	0.00187	0.04524	0.03417	0.18275
**OTU_127**	*Clostridium* XlVb spp.	1.000	0.00046	0.05721	0.00046	0.02227	0.03430	0.18275
**OTU_101**	Unclassified Porphyromonadaceae	0.938	0.06070	0.03301	0.00996	0.00637	0.03494	0.18280
**OTU_9**	*Oscillibacter* spp.	0.922	2.84601	4.30590	0.35882	0.51769	0.03583	0.18417
**OTU_99**	*Acetatifactor* spp.	1.000	0.10752	0.02289	0.03348	0.00848	0.03667	0.18524
**OTU_112**	*Catabacter* spp.	1.000	0.07594	0.01002	0.02684	0.00264	0.03974	0.19737
**OTU_157**	Unclassified Lachnospiraceae	1.000	0.00233	0.02214	0.00157	0.00809	0.04090	0.19972
**OTU_28**	*Acetatifactor* spp.	1.000	1.03943	0.51167	0.21719	0.06278	0.04348	0.20746
**OTU_71**	*Acetatifactor* spp.	0.752	0.01292	0.16159	0.00738	0.06235	0.04459	0.20746
**OTU_66**	*Barnesiella* spp.	1.000	0.01277	0.39069	0.00431	0.15916	0.04494	0.20746
**OTU_122**	Unclassified Lachnospiraceae	1.000	0.00283	0.05757	0.00071	0.02310	0.04532	0.20746
**OTU_51**	Unclassified Ruminococcaceae	0.954	0.16595	0.25148	0.01127	0.03589	0.04741	0.21369
**OTU_85**	Unclassified Lachnospiraceae	1.000	0.04245	0.09373	0.00814	0.02157	0.04970	0.21825
**OTU_103**	Unclassified Lachnospiraceae	1.000	0.00701	0.08933	0.00220	0.03566	0.04991	0.21825

Mice were fed a WD or a WD + vildagliptin (WD+V). Data were analysed using a Welch's *t* test and *p* value was corrected with the Benjamini-Hochberg method. Differences were established at q<0.05.

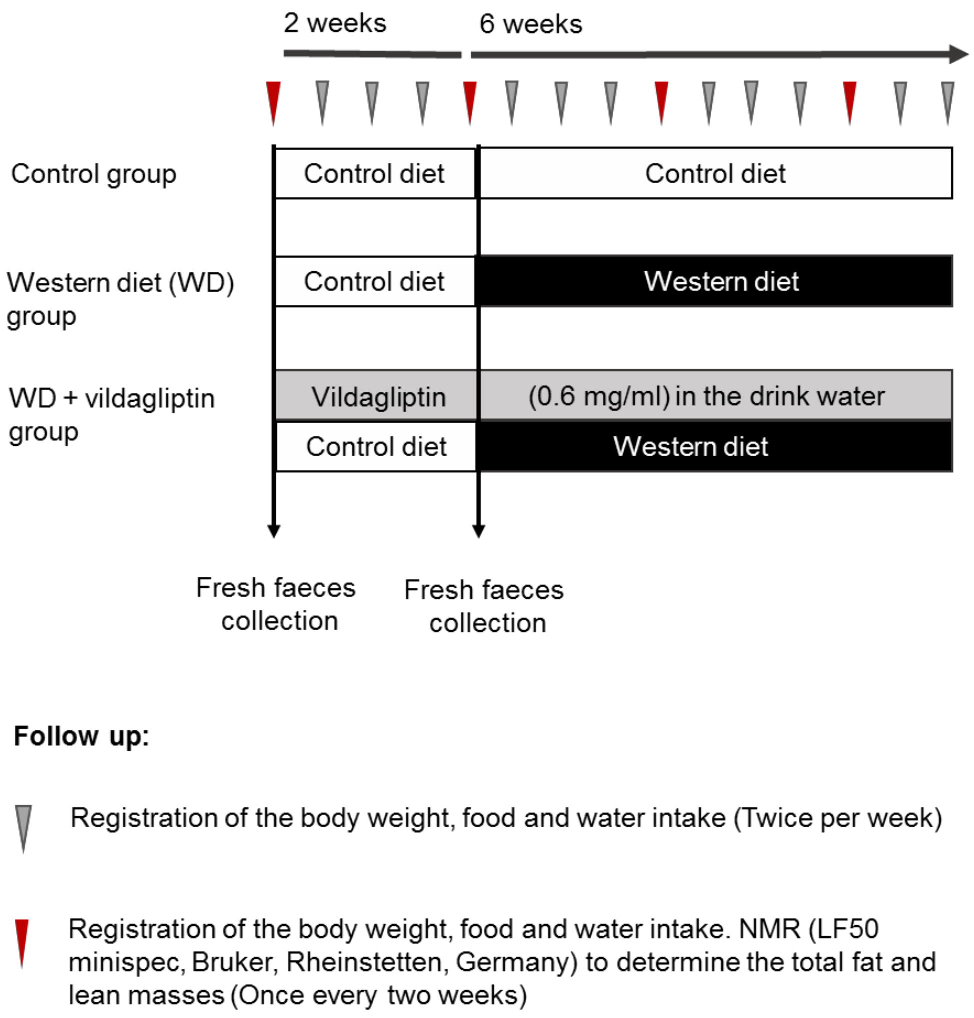

ESM Fig. 1: Scheme of the experimental design**.** Mice were fed a control diet, a WD or WD + vildagliptin. Vildagliptin was administered for two weeks, and co-administered with the WD until the end of the experiment (8th week).

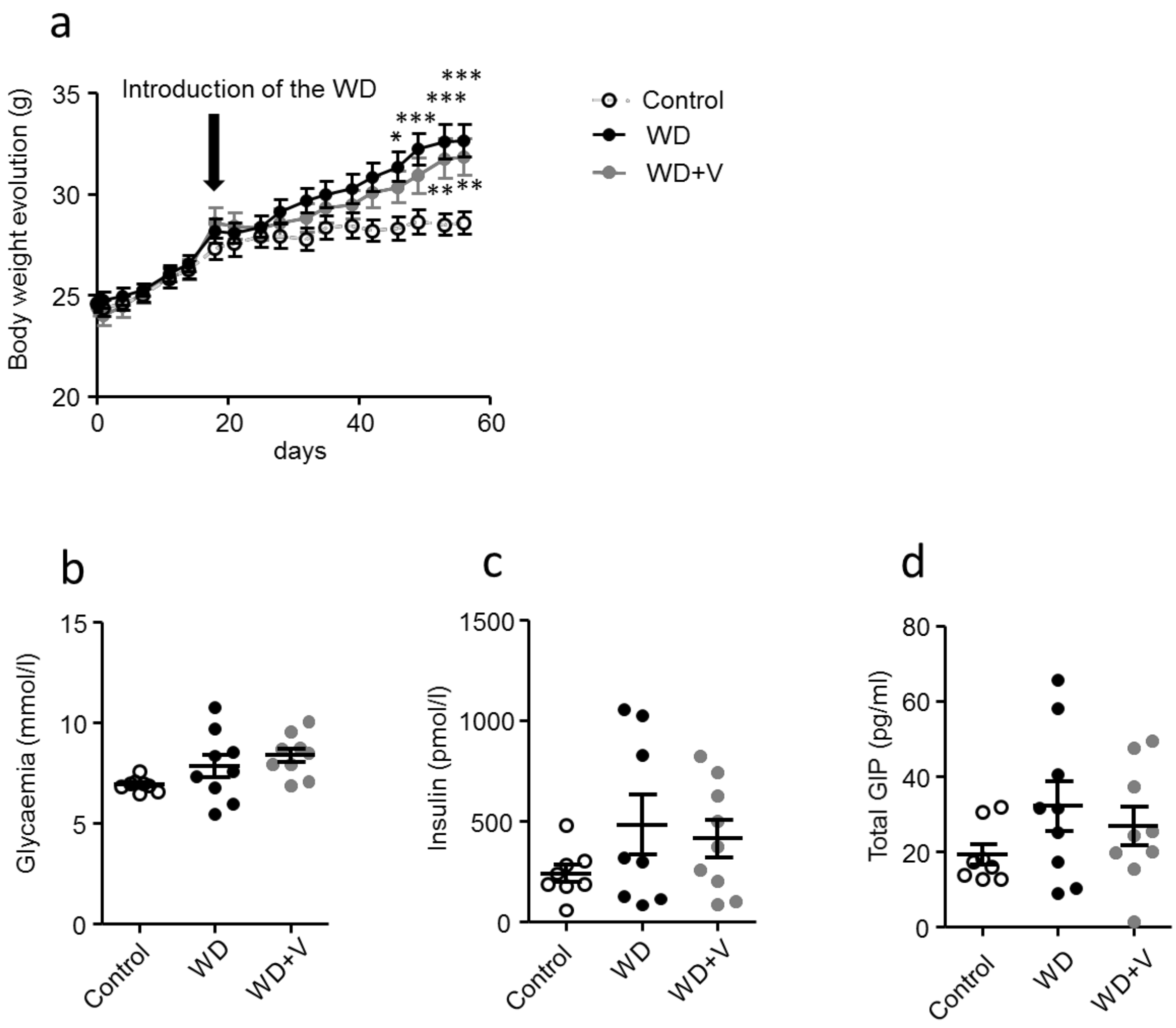

ESM Fig. 2: (a) Body weight evolution, **(b)** glycaemia after 6-hour of fasting, **(c)** insulin and, **(d)** total GIP concentrations in the portal vein. Mice were fed a control diet, a WD or a WD + vildagliptin (WD+V). Data of body weight evolution was analysed using two-way ANOVA followed by Bonferroni's post hoc test. Significant differences versus the control group are represented as * p<0.05, ** p<0.01, *** p<0.001. Glycaemia, insulin and, total GIP concentrations were analysed using one-way ANOVA followed by Tukey's post hoc test.

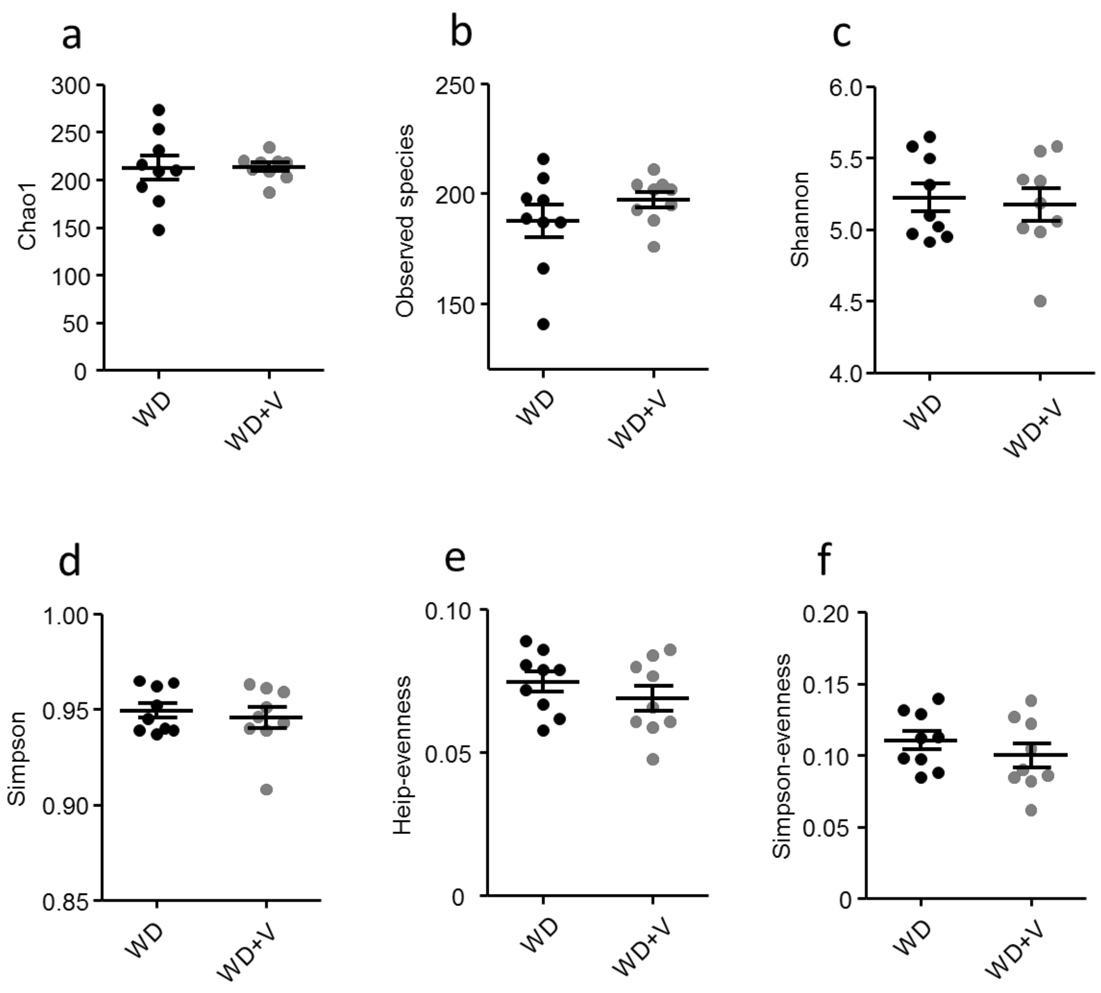

ESM Fig. 3: (a) Chao1, **(b)** observed species, **(c)** Shannon, **(d)** Simpson, **(e)** Heip-evenness, and **(f)** Simpson-evenness. Mice were fed a WD or a WD + vildagliptin (WD+V). Data were analysed using a Welch's *t*-test.

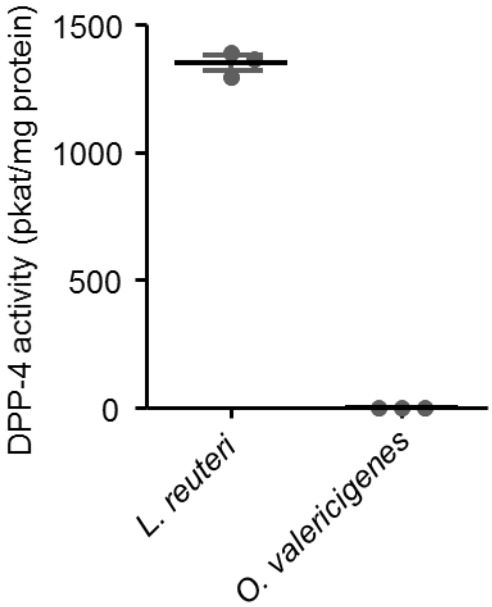

ESM Fig. 4: DPP-4 activity in the cell-free extracts of *L. reuteri* and *O. valericigenes*. No statistical analyses could be performed as *O. valericigenes* presented no DPP-4 activity.

a

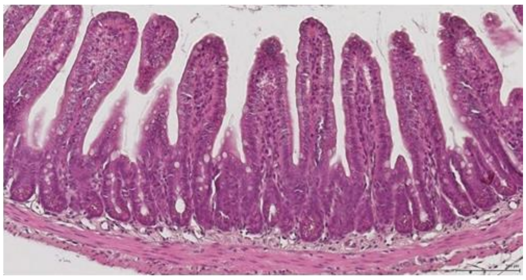

Control

b

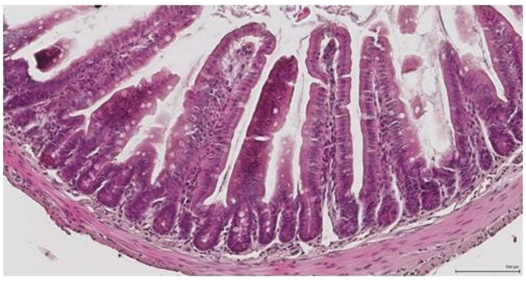

Western diet

c

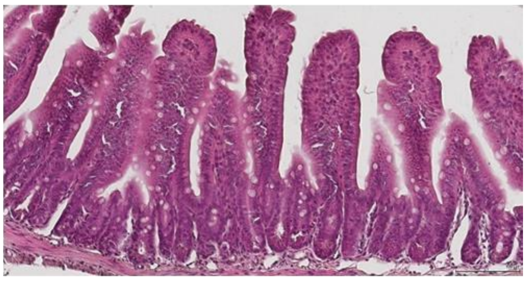

Western diet + vildagliptin

ESM Fig. 5: Representative H&E stained pictures of ileum of mice fed **(a)** a control diet, **(b)** a Western diet, and **(c)** Western diet + vildagliptin.

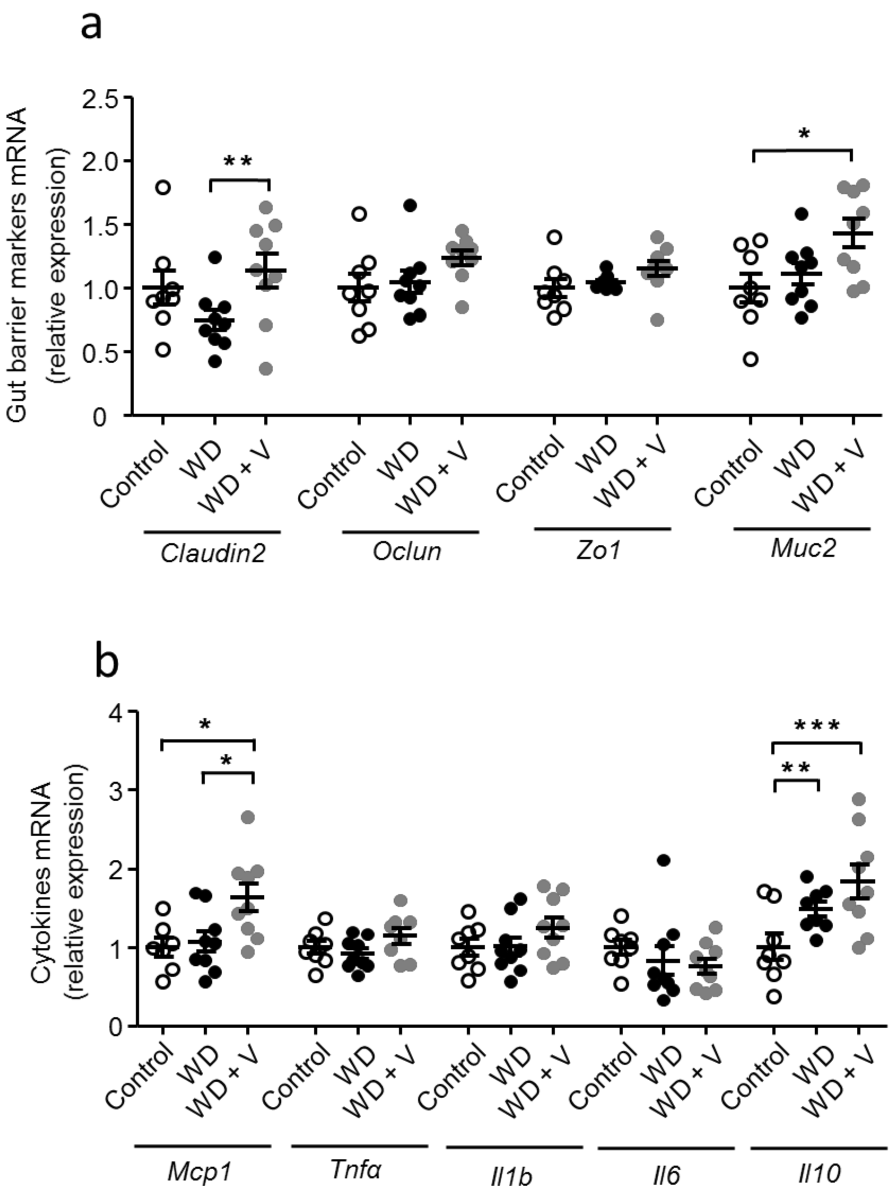

ESM Fig. 6: Effect of vildagliptin in **(a)** markers of the gut barrier function, and **(b)** inflammation in the ileum. Mice were fed a control diet, a WD, or a WD + vildagliptin (WD+V). Significant differences between conditions are represented as * p<0.05, ** p<0.01, *** p<0.001 according to the one-way ANOVA followed by Tukey's post hoc test.

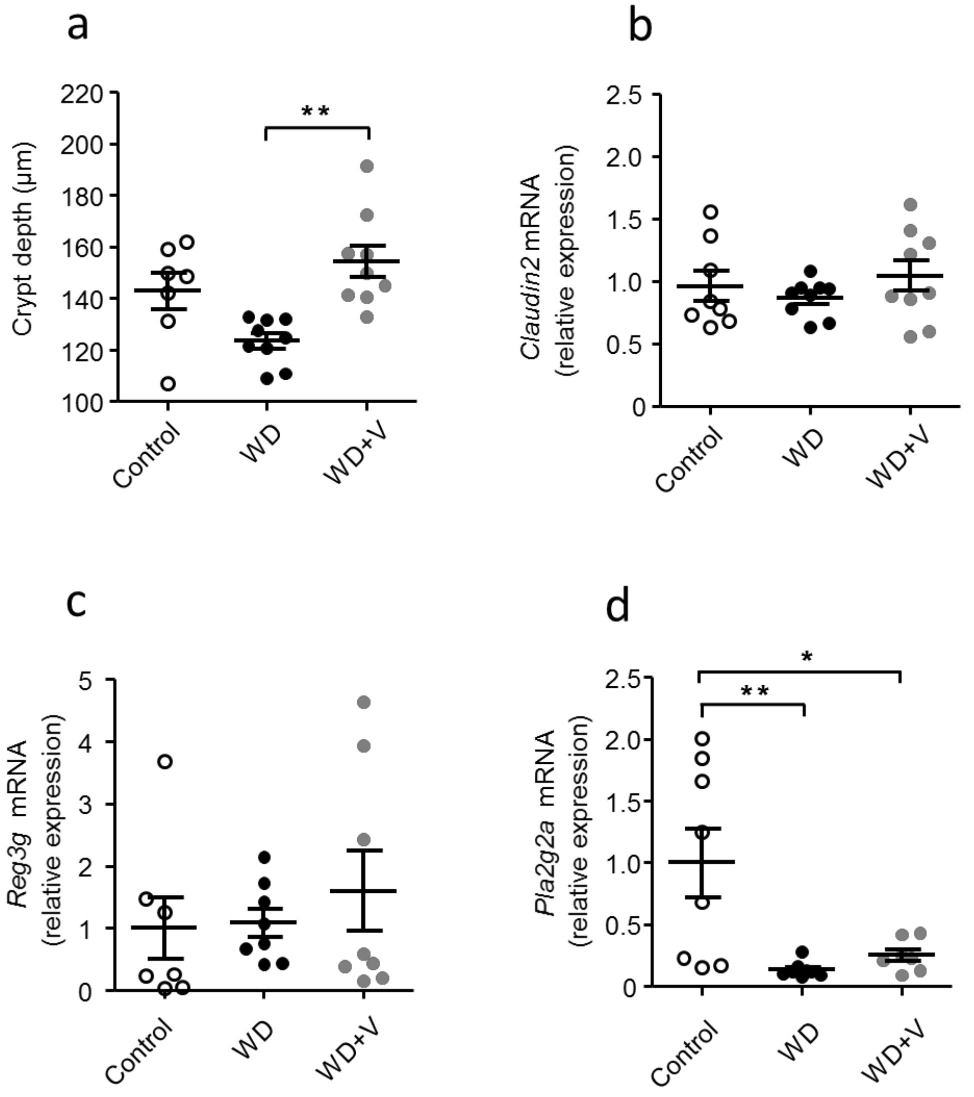

ESM Fig. 7: (a) Crypt depth, and gene expression of **(b)** *Claudin2*, **(c)** *Reg3g* and, **(d)** *Pla2g2*a. Mice were fed a control diet, a WD, or WD + vildagliptin (WD+V). Significant differences between conditions are represented as * p<0.05, ** p<0.01, according to the one-way ANOVA followed by Tukey’s post hoc test.

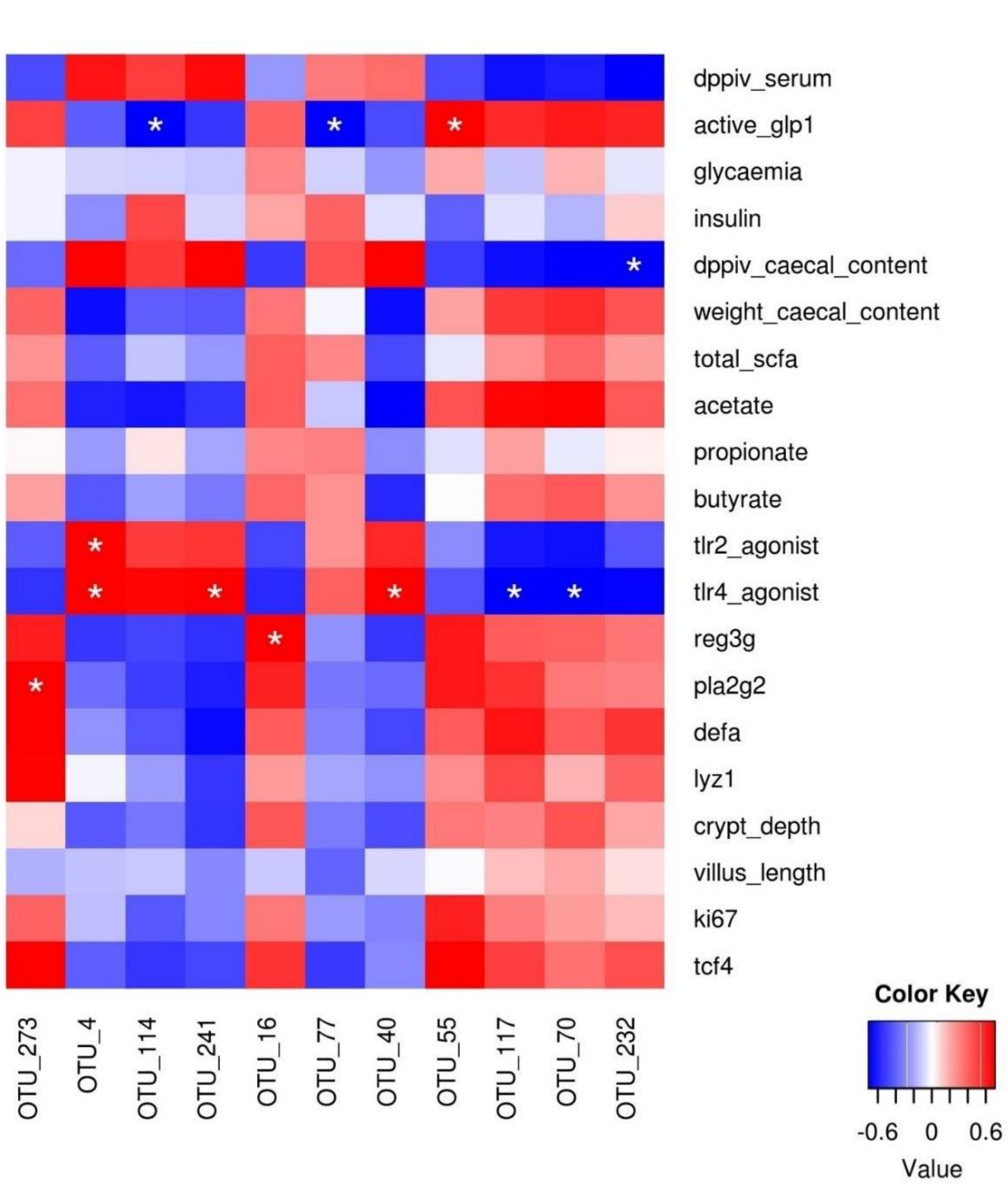

ESM Fig. 8: Heat map representation of Spearman correlation coefficients between OTUs and host parameters. *P* values were adjusted for multiple testing according to the Bonferroni-Hochberg procedure. Only OTUs for which at least one significant correlation with a host parameter was detected is displayed. The color at each intersection refers to the value of the rho coefficient. A significant correlation between two parameters at $p<0.05$ is represented by the symbol *. OTUs have been identified as follow, with the identity score within brackets: OTU 273, *Barnesiella* spp. (0.941); OTU 4, *Oscillibacter* spp. (1.000); OTU 114, *Acetatifactor* spp. (1.000); OTU 241, unclassified Ruminococcaceae (0.941); OTU 16, unclassified Porphyromonadaceae (1.000); OTU 77, unclassified_Lachnospiraceae (1.000); OTU 40, Catabacter spp. (0.971); OTU 55, Alistipes spp. (1.000); OTU 117, unclassified Lachnospiraceae (1.000); OTU 70, *Parabacteroides goldsteinii* (1.000); OTU 232, unclassified Lachnospiraceae (0.971).

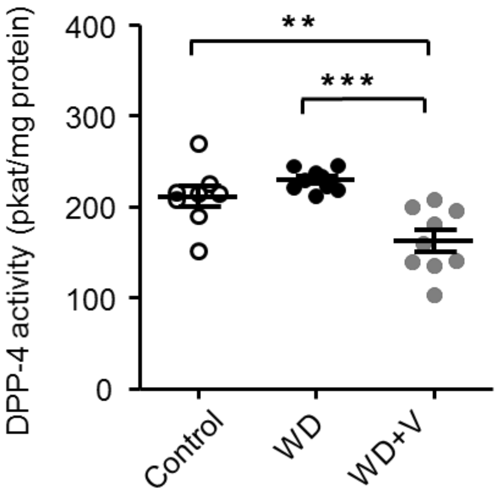

ESM Fig. 9: DPP-4 activity in the liver. Mice were fed a control diet, a WD or WD + vildagliptin (WD+V). Significant differences between conditions are represented as ** *p*<0.01, *** *p*<0.001 according to the one-way ANOVA followed by Tukey's post hoc test.
